# A deeper consideration of sex/gender in quantitative health research: a checklist for incorporating multidimensionality, variety, embodiment, and intersectionality throughout the whole research process

**DOI:** 10.1186/s12874-024-02258-7

**Published:** 2024-08-10

**Authors:** Christina Hartig, Sophie Horstmann, Katharina Jacke, Ute Kraus, Lisa Dandolo, Alexandra Schneider, Kerstin Palm, Gabriele Bolte

**Affiliations:** 1 Institute of Public Health and Nursing Research, Department of Social Epidemiology, University of Bremen, Bremen, Germany; 2 Health Sciences Bremen, University of Bremen, Bremen, Germany; 3 Gender and Science Research Unit, Institute of History, Humboldt-University of Berlin, Berlin, Germany; 4 German Research Center for Environmental Health, Institute of Epidemiology, Helmholtz Zentrum München, Neuherberg, Germany

**Keywords:** Sex, Gender, Sex/gender, Multidimensionality, Variety, Embodiment, Intersectional framework, Guideline, Checklist, Research process

## Abstract

**Background:**

There is a growing awareness of the need to adequately integrate sex and gender into health-related research. Although it is widely known that the entangled dimensions sex/gender are not comprehensively considered in most studies to date, current publications of conceptual considerations and guidelines often only give recommendations for certain stages of the research process and - to the best of our knowledge - there is a lack of a detailed guidance that accompanies each step of the entire research process. The interdisciplinary project “Integrating gender into environmental health research” (INGER) aimed to fill this gap by developing a comprehensive checklist that encourages sex/gender transformative research at all stages of the research process of quantitative health research. In the long term this contributes to a more sex/gender-equitable research.

**Methods:**

The checklist builds on current guidelines on sex/gender in health-related research. Starting from important key documents, publications from disciplines involved in INGER were collected. Furthermore, we used a snowball method to include further relevant titles. The identification of relevant publications was continued until saturation was reached. 55 relevant publications published between 2000 and 2021 were identified, assessed, summarised and included in the developed checklist. After noticing that most publications did not cover every step of the research process and often considered sex/gender in a binary way, the recommendations were modified and enriched based on the authors’ expertise to cover every research step and to add further categories to the binary sex/gender categories.

**Results:**

The checklist comprises 67 items in 15 sections for integrating sex/gender in quantitative health-related research and addresses aspects of the whole research process of planning, implementing and analysing quantitative health studies as well as aspects of appropriate language, communication of results to the scientific community and the public, and research team composition.

**Conclusion:**

The developed comprehensive checklist goes beyond a binary consideration of sex/gender and thus enables sex/gender-transformative research. Although the project INGER focused on environmental health research, no aspects that were specific to this research area were identified in the checklist. The resulting comprehensive checklist can therefore be used in different quantitative health-related research fields.

**Supplementary Information:**

The online version contains supplementary material available at 10.1186/s12874-024-02258-7.

## Background

There is a growing awareness of the need to integrate sex and gender adequately into health-related research [1–5]. For appropriate integration to be successful, the definitions of sex and gender, their multidimensionality and their underlying concepts must be known and applied correctly [6]. In the concepts currently discussed in health-related research, the term sex as a multidimensional biological construct refers to attributes such as anatomy, physiology, genes and hormones and is usually operationalised as female, male or intersex [2, 5–7]. Gender as a multidimensional social construct refers to roles, behaviours, relationships, power relations and other aspects that are usually socially associated with certain gender identities [2, 6, 7]. Thus, gender does not only act on an individual but on a structural and symbolic level as well [8] (see glossary). Both the sex-linked biology and gender refer to multiple dimensions that are entangled but, however, not mutually dependent [2, 9]. The variety of sex/gender cannot be accurately captured by a dichotomous understanding of male/female. Thus, there is a need for new approaches that adequately capture this complexity of sex/gender and its diversity beyond binary categories [3, 9–11]. To highlight the entanglement of sex and gender, we use the term sex/gender for a non-binary category, that has multiple, interwoven biological and social dimensions, as it is also conceptualised in the embodiment theory [2, 12, 13] (see glossary). An intersectionality perspective, which means considering the intersection of sex/gender with other social categories (e.g. socioeconomic position, race/ethnicity, sexual orientation) and the associated privileges and oppressions, strengthens the consideration of structural causes of health inequities such as systems of power and discrimination processes [2, 14–17] (see glossary).

Sex/gender-equitable research profoundly includes the needs of all sex/gender groups throughout the entire research process [3] (see glossary). Adopting an intersectionality perspective enables the recognition, reflection, and consideration of connections and interactions of sex/gender with multiple social categories, power relations, as well as possible discrimination structures and processes and can thus lead to a more sophisticated differentiation of sex/gender groups [2, 15, 18]. Highlighting gendered power relations, norms, roles and stereotypes in research might help to reduce sex/gender discrimination and health-related sex/gender differences resulting from inequities and structural disadvantage [3].

**Fig. 1:**
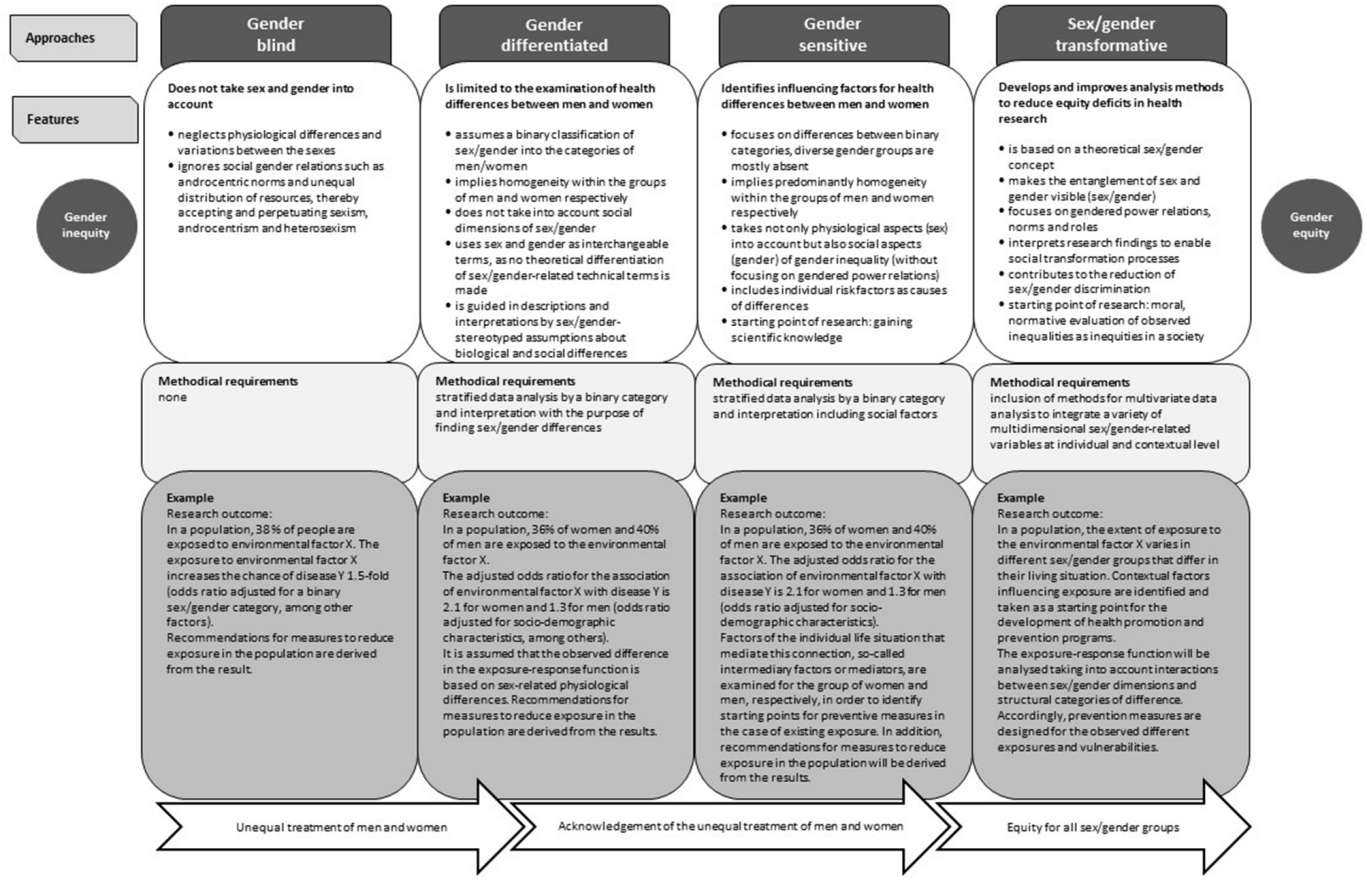
Conceptual differences of approaches to take sex/gender into account in quantitative health research [Based on the framework of Pederson et al., 2014, further adapted to epidemiological research]

Not all approaches to include sex/gender into health-related research are sex/gender-equitable. Different approaches and their features and methodological requirements can be seen in Fig. 1. A gender-blind approach can contribute to unequal treatment of men and women, while the gender-differentiated approach takes into account sex differences and the gender-sensitive approach additionally considers biological and social factors that lead to health-related differences between sex/gender groups. However, these three approaches cannot remove inequalities between women and men, only the fourth sex/gender-transformative approach leads to equity of all sex/gender groups. Sex/gender transformation aims to address the root causes of sex/gender inequities and works to change harmful gendered power relations, norms, roles and stereotypes [19, 20] (see glossary). There may be further approaches to integrate sex/gender in health-related research that are not explicitly shown in the figure. When using the framework researchers should consider that in practice, extreme or mixed forms of the presented approaches may occur.

## Objective

Although it is widely known that sex/gender is not comprehensively considered in most studies to date [4, 6, 21, 22], current publications of conceptual considerations and guidelines often contain recommendations that refer to individual phases of the research process. To the best of our knowledge, there is lack of detailed guidance that accompanies the adequate consideration of sex/gender concerning multidimensionality, variety, embodiment and intersectionality in each step of the entire research process. So, the aim of this publication is to raise awareness and to provide assistance in form of a checklist that encourages sex/gender transformative research at all stages of the research process, focussing on quantitative health research.

## Methods

### INGER project

This checklist was developed as part of the collaborative research project INGER (“Integrating gender into environmental health research”). INGER focuses on improving research by integrating sex/gender into environmental health research and thus aims to make it more sex/gender-equitable (https://www.uni-bremen.de/en/inger). As part of the project, systematic reviews were conducted, which showed that sex/gender has not yet been adequately considered in environmental health research [23, 24] and Bolte et al. developed a multidimensional sex/gender concept including intersectionality [2]. Furthermore, an operationalisation of the developed concept was introduced [25] and innovative methods for analysing data on sex/gender in population-based environmental health studies were implemented [26].

### Development process

The checklist presented in this publication builds on current guidelines promoting the adequate consideration of sex/gender from an intersectional perspective in health-related research. Starting from three important key documents “Gendered Innovations” [27], “Gendered Innovations 2” [22] and the “SAGER Guidelines” [4], further relevant publications were retrieved. Firstly, we collected publications that are currently consulted within the disciplines involved in INGER (epidemiology, public health, gender studies and psychology). Furthermore, we used a snowball method by searching for further relevant titles in the bibliographies of the publications that were already included. Since our checklist was intended to refer to current approaches, our selection was limited to papers published between 2000 and 2021. We restricted our search to documents written in English or German. The identification of relevant publications was continued until saturation was reached.

55 relevant publications published between 2000 and 2021 were identified and included in the development of this checklist (see supplement). In a first step, recommendations referring to sex/gender consideration in the research process were extracted from these 55 publications, partly using words close to the original text, assigning them to the respective section of all steps of the research process as given below. The collection and review of the publications was carried out by two members of the research team.

Steps of a research process:

- background assumptions and theory,
- scientific evidence base,
- research questions and hypotheses,
- study design,
- study population,
- operationalisation of sex/gender,
- data collection,
- data analyses,
- presentation of results,
- interpretation/discussion,
- publication/dissemination,
- research team,
- transfer,
- science and risk communication,
- avoidance of stereotypes and.
- language.

In a second step, the contents were summarised in a condensed form, as some publications gave the same or similar recommendations. As the recommendations of previous publications did not cover every step of the research process and often considered sex/gender in a binary way, we filled this research gap in a third step. We modified and enriched the recommendations based on the expertise of the researchers of the INGER study group, aiming to add further categories to the binary sex/gender categories by considering sex/gender from an intersectional perspective. We do not claim that the developed checklist is complete.

## Results

The checklist addresses sex/gender aspects of the whole research process of planning, implementing and analysing quantitative health studies as well as aspects of appropriate language, communication of results to the scientific community and the public, and composition of the research team. It comprises 67 sex/gender related items in 15 sections, key points that refer to an intersectional approach to sex/gender were marked (Table 1). A glossary provides a compact overview of the most important terms (Table 2).

**Table 1:** Comprehensive checklist for the consideration of sex/gender [4, 6, 7, 10, 13, 14, 18, 21, 22, 27–72]

Checklist								
**1. Background assumptions and theory**								
**-**	Check the relevance and conceptualisation of sex/gender for your research project.							
	• Consider whether sex/gender is probably an explanatory factor, an effect modifier or merely a confounder. • Clarify the way sex/gender might act and which dimensions of sex/gender (biological or social) could be important (a causal diagram may help making assumptions). • If possible, consult with different sex/gender groups in the study population to identify sex/gender-related issues.							
**-**	Give a precise definition of all terms used (“sex”, “gender”, “sex/gender”) and their underlying concepts.							
	• The terms sex and gender should not be confused or used interchangeably or as proxies for each other. • The term “sex/gender” may be used to indicate the entanglement of sex and gender and should be explained in this regard.							
**-**	Avoid perpetuating confusion and inaccuracies. Consider what opportunities have been missed in the past as a result of failing to analyse sex/gender accurately and comprehensively.							
**-**	Use an intersectional approach to include multiple identities, social positions and intersecting processes. Keep in mind that sex/gender does not operate in isolation but intersects with other social determinants of health.							***intersectionality***
2. **Scientific evidence base**								
**-**	If applicable use sex/gender specific search strategies for the identification of scientific evidence. Be aware of using several search terms to capture the evidence in the existing literature. Sex/Gender issues might be masked by overarching terms such as social determinants of health or individual characteristics.							
**-**	Review existing literature about the topic of interest with regard to the relevance of several dimensions of sex/gender (e.g. sex assigned at birth; current sex/gender identity; internalized sex/gender roles; externalized sex/gender expressions). Be aware of the sometimes misleading use of terms “sex” and “gender” in current literature.							
**-**	Give a rationale within your introduction whether sex/gender impacts - in terms of exposure variation, effect modification, direct effect on the outcome or confounding - may be expected based on previous scientific evidence or theory-based assumptions.							
3. **Research questions and hypotheses**								
**-**	Formulate your research question appropriately depending on whether exploratory or confirmatory research is planned. If applicable, include a testable hypothesis focusing on sex/gender.							
**-**	Make sure to formulate your research question and hypothesis sex/gender-equitable. Indicate how sex/gender can be of relevance.							
**-**	Make sure that your research question is applicable to different sex/gender groups from an intersectionality perspective (see glossary). If this is not reasonable indicate why it only applies to a specific group.							***intersectionality***
4. **Methods**								
**4.1 Study design**								
**-**	Consider which data sources are suitable for your research question and plan your study accordingly: e.g. primary data, secondary analyses of existing research data or secondary data (not originally collected for research purposes and possibly inadequate in terms of multiple sex/gender dimensions). • Always report where the data come from and how they were collected. State also if no details are known.							
**-**	Incorporate dimensions of sex/gender (e.g. sex assigned at birth; current sex/gender identity; internalized sex/gender roles; externalized sex/gender expressions) into the design of your research project wherever possible or appropriate so that the most important sex/gender aspects can be adequately addressed. • Report how sex/gender is taken into account within your study design and methodology and justify your decision. • Give a rationale for the inclusion or exclusion of sex/gender, respectively, and if applicable why no sex/gender analysis was conducted. ➝ The absence of previous evidence does not, by itself, constitute justification to not include sex/gender into data collection, analysis and interpretation of results or to only study a specific sex/gender group.							
**-**	Consider incorporating a mixed method design to get deeper insights into sex/gender-related aspects.							
**-**	Plan to analyse gendered phenomena on several levels (structural, group, individual).							
	• Identify the principal processes or practices that are producing the gendered phenomena on the three levels.							
	• Examine overlaps between and variations within groups of different sexes/gender.							
**-**	Describe any efforts to address potential sources of gender bias like ignoring or overemphasising sex/gender differences.							
**-**	Develop a data analysis plan that includes sex/gender prospectively.							
	• Make sure to design your research in a way that sex/gender impacts concerning exposure variation, effect heterogeneity or differences in the health outcome can be revealed.							
	• When conducting studies only on one sex/gender group, take the heterogeneity within this group into account. → In case of topics and questions which pertain to all sex/genders, a justification is required if only a specific sex/gender is included.							
**-**	In longitudinal research, consider how changes at the contextual level may affect the cohort under investigation. Examine how the observed biological and social sex/gender dimensions change and evolve over time (e.g. gender identity, gender roles and care activities). These dimensions should therefore be measured more often throughout follow-up, not only once at baseline.							
**4.2 Study population**								
**-**	Justify the selection of the population groups to be studied in view of the research question.							
**-**	Make sure that the size of the study population and subgroups are sufficient to maintain statistical power and to detect differences between sex/gender groups, if they exist.							
**-**	Ensure that the study population is appropriately composed in terms of biological and social dimensions of sex/gender.							
	• If possible and appropriate, take into account further sex/ gender-related information when deciding upon the study population. These may concern biological or social aspects such as gendered experiences or attitudes.							
	• Consider that there is heterogeneity within a group of individuals of the same sex/gender.							
**-**	If the study design includes randomisation, then consider multiple sex/gender groups from an intersectionality perspective. If the study population was randomised by sex/gender and so conceptually is taken into account as a confounder, keep in mind that no further sex/gender analyses can be conducted (e.g. test for effect modification).							
**4.3 Operationalisation of sex/gender**								
**-**	Variables of interest should be operationalised according to existing standards whenever possible. • Decide which sex/gender dimensions may be of interest to answer your research question and choose specific and appropriate variables to measure them. • Depending on your research question it might be appropriate to use single variables for each sex/gender dimension or to apply an aggregate measure (e.g. gender index, score).							
	• If only one binary variable is available from the survey, the sex/gender variable in your research could be named based on what it actually contains: If it is the self-reported sex/gender, it tends to show a person’s gender identity, while population registers predominantly show the sex assigned at birth.							
**-**	Think beyond a binary understanding of sex/gender and make sure to consider the multidimensionality of biological and social dimensions of sex/gender.							
	• Use more comprehensive and precise survey-based tools to measure sex/gender: make sure that all characteristics and expressions are precisely defined (uniqueness), that each respondent falls exactly into one of the given variable expressions (exclusivity) and that every possible characteristic expression is covered (exhaustivity).							
	• If your research question addresses specific biological sex/gender factors (such as e.g. pregnancy or susceptibility to prostate cancer) or specific social sex/gender factors (such as e.g. attitudes, needs and interests) they should be operationalised directly and not summarized under an unspecific sex/gender term.							
**-**	If applicable, integrate individual-level aspects (e.g. sex assigned at birth, current sex/gender identity, internalised sex/gender roles, externalized sex/gender expressions) and contextual-level aspects (e.g. discrimination experiences, structural disadvantages, societal power relations).							
**4.4 Data collection**								
**-**	When collecting data, use a sex/gender-equitable approach in regard of the collected content and the way the data collection is conducted.							
**-**	Decide which information to collect based on the prior literature review. Make sure that the information is relevant and not just based on “well-known” delusions.							
**-**	Report how information on sex/gender was obtained.							
	• For each variable of interest, give sources of data and details of methods of assessment (measurement). Describe comparability of assessment methods if there is more than one.							
	• The measurement properties, including validity and reliability, of the instruments used should be presented (e.g. according to sex/gender).							
**-**	Use for data collection and measurements whenever possible only instruments which are valid and reliable in all sex/gender groups. → It may be necessary to use alternative instruments. • If they exist, standardised terms should be used to address all sex/gender groups. • Remember that different issues can apply in different ways to different sex/gender groups. Consider all relevant variables and themes to give validity to this.							
**-**	Bear in mind that in case of sensible data, participants may feel more comfortable filling out a questionnaire rather than answering questions in an interview.							
**-**	Make sure that data is collected in a non-discriminating way.							
**-**	Consider power inequities during data collection. They may affect the participation.							
**-**	Choose a convenient time and place in which to engage in data collection.							
**4.5 Data analyses**								
**-**	Make sure to use an appropriate and sex/gender-equitable methodology to analyse your data: • Examine your results systematically by sex/gender. Especially by those sex/gender dimensions, which have been considered as relevant for the research question. • Use the same methodology or approaches for every included sex/gender group. Give a rationale if you are treating specific groups differently. • If your research does not focus specifically on sex/gender, take possible effect modification by sex/gender into account in the analysis. • If your research does not focus specifically on sex/gender, do not control for sex/gender by default in the analysis: only do so if sex/gender has been identified as a confounder. • To assess possible selection effects according to sex/gender, a stratified analysis of the response is necessary. • In terms of methods and content considerations: decide what kind of sensitivity analyses might be useful.							
**-**	Prespecify the methods you are going to apply and report and justify if tests were applied post hoc.							
**-**	Try to replicate your findings if possible.							
**-**	If possible, directly test for the underlying mechanisms.							
**-**	Ensure an appropriate intersectional methodology:						***intersectionality***	
	• Identify other intersecting characteristics carefully by using theoretical evidence and community knowledge. It might help to refer to the framework “PROGRESS-Plus” to remember that sex/gender can be influenced by or interact with: place of residence, race/ethnicity/culture/language, occupation, religion, education, socioeconomic position, social capital, personal characteristics associated with discrimination (e.g. age, disability), features of relationships (e.g. social support) and time-dependent relationships (e.g. leaving the hospital, respite care, other instances where a person may be temporarily at a disadvantage).							
	• Use appropriate and valid measures across all intersections.							
	• Use appropriate methods for the analyses of complex interactions.							
	• Analyse the economic, political and cultural contexts in which the phenomena occur in order to understand the major underlying forces that motivate and sustain the practices in question.							
**-**	You may revisit already existing analysis that missed taking sex/gender into account and perform a sex/gender informed secondary analysis.							
5. **Presentation of results**								
**-**	Report data in a sex/gender-equitable way.							
	• Present number and results disaggregated by sex/gender. • Report the sex/gender of your subjects, even in single-sex/gender studies.							
	• For each variable of interest report effect sizes and number of participants with missing data.							
	• Report “null” findings.							
**-**	In clinical trials, data on withdrawals and dropouts should also be reported disaggregated by sex/gender.							
**-**	Be aware of the sex/gender of all those involved in the research process, including the research team, and mention if it is relevant to or has an impact on the conduct and analysis of the study.							
**-**	Report if data on sex/gender is not available and if it is not known how data on sex/gender was collected and operationalised.							
**-**	Justify why any planned analyses were not conducted.							
**-**	Rethink your visual presentation. Ensure a balanced presentation of sex/gender results.							
	• Establish a balance in considering all sex/gender groups in the presentation of results (e.g. response rate, exposure and outcome proportions, effect estimates).							
	• To communicate your results, use language, pictures, symbols and examples that include diverse groups, e.g., people with disabilities, lesbian and gay parents with their families, different ethnicities.							
6. **Interpretation / Discussion**								
**-**	Discuss the potential implications of several dimensions of sex/gender on the study analyses and results.							
**-**	Consider sex/gender dimensions (e.g. sex assigned at birth; current sex/gender identity; internalized sex/gender roles; externalized sex/gender expressions) within the interpretation of your results: Refer to gender-theoretical concepts (e.g. variety, multidimensionality, embodiment and intersectionality) and interpret sex/gender differences in the light of biological plausibility, social context, processes of embodiment and power relations.							
**-**	If sex/gender was relevant for your research question, give a rationale if no sex/gender analysis was conducted and discuss the implications.							
**-**	Discuss limitations of the study and take into account sources of potential bias or imprecision. Discuss both direction and magnitude of any potential bias related to sex/gender.							
**-**	Avoid overemphasizing sex/gender differences.							
**-**	Avoid generalization. Avoid using one sex/gender group as norm for everybody or present results from one group as applicable to all “subjects”.							
**-**	Present sex/gender specific recommendations.							
**-**	Outline research gaps or unanswered questions related to sex/gender analyses, and identify planned analyses that could not be conducted due to unavailable data.							
**-**	Remove assumptions that may limit or restrict innovation and knowledge in unconscious ways. Remove assumptions that unconsciously reinforce gender inequalities.							
**-**	Do not confuse given facts with opinions and attitudes towards certain sex/gender groups.							
**-**	Describe to whom the available evidence applies in relation to sex/gender and any intersecting characteristics and interpret findings on a structural level (societal context, power relations).							***intersectionality***
7. **Publication / Dissemination**								
**-**	Disseminate results in a sex/gender-equitable way.							
	• Avoid underreporting by disseminating all of your results even if your findings suggest that sex/gender has no impact.							
	• Consider publishing your study protocol in advance to avoid publication bias.							
	• Specify the particular sex/gender group that was the focus of the study within title and abstract.							
	• If central to the research question, present sex/gender findings and implications for further research and practice in the abstract.							
**Research team**								
**-**	Choose the composition of the research team carefully and ensure a high level of diversity between team members and additionally consider using a participative research approach to include opinion and experiences of further groups.							
	• Diverse working groups usually have a positive impact on the quality of science due to the variety of perspectives, experiences and skills of their members.							
	• Be constantly aware of your and your team’s own sex/genders, biases, preferences, values and socio-cultural background and keep in mind that these factors might influence the research process and its findings.							
**-**	Ideally work together with experts from the field of sex/gender research or alternatively consider taking part in an expert lead sex/gender training before conducting your research.							
**Transfer, science and risk communication**								
**-**	Convey the results to the affected population groups and other actors (e.g. social partners, politicians, self-governance organisations) in easy-to-understand language and choose suitable means of access and formats to do so.							
**-**	Use a range of culturally relevant strategies to communicate your results, for example, community print, radio and television, the Internet, presentations in community languages to relevant groups.							
**Avoidance of stereotypes**								
**-**	Avoid reproducing stereotypes during the whole research process.							
-	Treat every subgroup with the same respect aiming for more equity.							
**Language**								
**-**	Make sure to use terms or grammar equally for different sex/gender groups.							
-	Use gender impartial language when you are talking about different sex/gender groups.							
**-**	Only use gender specific terms, when you are talking about a particular sex/gender group.							
**-**	Avoid using a derogating or discriminating language.							
**-**	Reflect on how wording or messages can be tailored to sex/gender or other identity characteristics.							

## Discussion

So far, sex/gender has not often been comprehensively considered in health-related research, although it is known that adequate consideration of sex/gender together with potentially interacting social categories such as socioeconomic position, race/ethnicity and sexual orientation promotes scientific quality of the results. This may help to achieve more equity in health-related research in the long term. An adequate consideration of sex/gender can ensure that research results and prevention programs apply to all people in terms of sex/gender and do not only represent a certain part of society [4, 6, 21, 22].

The introduced checklist for the incorporation of sex/gender-related multidimensionality, variety, embodiment and intersectionality into the research process is a compilation of recommendations from 55 publications, identified from relevant publications of several disciplines. Recommendations were not only extracted and summarised, but also modified and enriched based on the interdisciplinary expertise of the authors in order to consider the complexity of sex/gender and go beyond a binary understanding.

Based on profound expert knowledge in the disciplines epidemiology, public health, and gender studies, and an already established collection of pertinent checklists, we selected three key documents for further search of checklists published in the last years. Instead of a systematic literature search in several literature databases with specific keywords, we used the snowball method until saturation in terms of recommendations given in the identified checklists was reached. This approach can be a limitation, because relevant literature might have not been identified in the development of our checklist. A great strength of our checklist is its comprehensiveness as it covers all steps of the research process. In this way, it represents a comprehensive aid for researchers. But at the same time this strength is also a limitation, as our aim of developing a comprehensive checklist has resulted in the final product being very complex. Thus, the presented items may not all be self-explanatory. In order to adequately use it, it might be necessary to include experts from the field of sex/gender research into the whole research process or to improve the sex/gender knowledge of members of the research team.

Although the INGER project focused on environmental health research, no aspects that were specific to this research area were identified in the checklist. To follow the recommendations given for evaluating the current scientific evidence base, further approaches of INGER, such as the sex/gender-focused systematic reviews [23, 24] or the newly developed matrix for evaluation of sex/gender consideration in epidemiologic studies [73], might be used.

## Conclusion

The developed comprehensive checklist goes beyond a binary consideration of sex/gender, includes complex dimensions of sex/gender, integrates a comprehensive consideration of gender-related power relations and thus encourages a sex/gender-transformative research approach.

**Table 2:** Glossary of the most important terms

	Glossary
Term	Definition
**Sex**	Sex is a multidimensional biological construct that refers to attributes, such as anatomy, physiology, genes, and hormones. In spite of the wide variation in the attributes that constitute sex, it is usually operationalised as female or male (or intersex). [2, 6, 7, 74]
**Gender**	Gender is a multidimensional social construct that refers to roles, behaviours, relationships, relative power, and other aspects that are usually socially associated with certain gender identities. It does not only act on an individual but on a structural and symbolic level as well: The individual level refers to how a person defines their own gender identity. The structural level describes how social relations are organised in terms of sex/gender in a certain group of people or the society as whole. These include, for example, the gendered division of labour and the existence of certain roles and norms that dictate what is generally considered appropriate for particular genders. The symbolic level reflects the process of ascribing gendered ideas to observations of the lived and inanimate environment, for instance the ascription of a gender and the characteristics associated with it to a car or natural phenomenon [2, 6–8]
**Sex/gender**	Sex and gender interact and are entangled. Therefore, they should not be considered separately. The term sex/gender stresses out this entanglement of the biological and social dimensions. [12, 13]
**Sex/gender-equitable**	The focus is on meeting the needs of all sex/genders, whether they are the same or different. Sex/Gender-equitable research highlights gendered power relations, norms and roles, contributing to the dismantling of sex/gender stereotypes, discrimination and unjust and avoidable sex/gender differences due to inequality and structural disadvantage. The implementation of sex/gender-equitable research therefore has a sex/gender-transformative effect. [3]
**Sex/gender-transformative**	A sex/gender-transformative approach promotes critical awareness of gendered power relations, norms and roles and highlights the effects of harmful and unjust power relations. In a next step, the sex/gender-transformative approach aims to address the root causes of sex/gender inequalities and works to change harmful gendered power relations, norms and roles. It demonstrates the advantages of changing harmful and unjust power relations with regard to health and social effects. [19, 20]
**Multidimensionality**	Sex and gender were each developed as concepts with several different dimensions. Sex refers to the biological characteristics for reproduction and to the embodied biological expression of gender. In the case of gender, the basis for the different dimensions are the individual, the structural and the symbolic levels. [2, 8, 9]
**Variety**	Sex/Gender cannot be adequately captured by a dichotomous understanding of male/female, as there is a wide variety in the attributes that constitutes both sex (as a biological construct that refers to genes, hormones, physiology, organs and anatomy) and gender (as a social construct that is culture-based, historically specific and therefore constantly changing). [2, 5, 47]
**Embodiment**	The term “embodiment” was defined by Krieger as “how we literally incorporate, biologically, the material and social world in which we live” (Krieger, 2005:352). Biological and social factors of sex/gender influence each other and therefore cannot be considered separately. To highlight this entanglement, as it is also conceptualised in embodiment theory, the term “sex/gender” could be used. [2, 9, 13, 75]
**Intersectionality**	Intersectionality is a theoretical framework that refers to the intersections of multiple social categories, power relations and processes of discrimination that are entangled with each other. The framework states that an individual is made up of many social positions and processes that interact with each other creating individual experiences that go beyond the mere addition of the single effects of these positions. Sex/Gender should therefore not be considered in isolation from other social identities. Instead, the heterogeneity within the sex/gender categories that is based on, for example, socioeconomic position, race/ethnicity, and/or sexual orientation should be taken into account. Intersectionality has its roots in black feminist scholarship with one of its most prominent representatives being Kimberlé Crenshaw. In the 1990s, she coined the term with her work on the marginalization of Black women. [2, 15, 16, 18]
**Power relations**	The concept of power relations refers to the distribution of power in a society. Inequalities arise from an unbalanced distribution of power between different groups of a population. Power relations decide over the roles, behaviours and attitudes that are considered appropriate for certain (sex/gender) groups and may lead to sanctions in case of violation. Simultaneously, those social norms can shape and reinforce the distribution of power. [5]

### Electronic supplementary material

Below is the link to the electronic supplementary material.

Supplementary Material 1

## Data Availability

The datasets generated and analysed during the current study is available from the corresponding author on reasonable request.
