# Supplementary material for: A deeper consideration of sex/gender in quantitative health research: a checklist for incorporating multidimensionality, variety, embodiment, and intersectionality throughout the whole research process
Source: BMC Med Res Methodol. 2024 Aug 10;24:180. doi: 10.1186/s12874-024-02258-7 (PMC11316289; doi:10.1186/s12874-024-02258-7)
Supplement: Supplementary file 1 — Supplementary Material 1 [file 12874_2024_2258_MOESM1_ESM.docx]

**A deeper consideration of sex/gender in quantitative health research: A checklist for incorporating multidimensionality, variety, embodiment, and intersectionality throughout the whole research process**

**Christina Hartig, Sophie Horstmann, Katharina Jacke, Ute Kraus, Lisa Dandolo, Alexandra Schneider, Kerstin Palm, Gabriele Bolte**

**Supplement: 55 publications included in the synthesis**

Bauer GR. Incorporating intersectionality theory into population health research methodology: Challenges and the potential to advance health equity. Social Science & Medicine. 2014; 110:10–7. doi:10.1016/j.socscimed.2014.03.022.

Bauer GR. Meet the Methods Series: Quantitative Intersectional Study Design and Primary Data Collection: Institute of Gender and Health. 2021. https://cihr-irsc.gc.ca/e/52352.html.

Bolte G. Gender in der Epidemiologie im Spannungsfeld zwischen Biomedizin und Geschlechterforschung. Konzeptionelle Ansätze und methodische Diskussionen. In: Hornberg C, Pauli A, Wrede B, editors. Medizin-Gesundheit-Geschlecht: Eine gesundheitswissenschaftliche Perspektive. Wiesbaden: Springer VS; 2016. p. 103–24 (Geschlecht & Gesellschaft; Band 55).

Bolte G, Lahn U. Geschlecht in der Public-Health-Forschung zu gesundheitlichen Ungleichheiten: Potenziale und Begrenzungen des Intersektionalitätsansatzes. GENDER – Zeitschrift für Geschlecht, Kultur und Gesellschaft. 2015; 7(2):51–67. doi:10.3224/gender.v7i2.19312.

BZPH. Zu mehr Gleichberechtigung zwischen den Geschlechtern: Erkennen und Vermeiden von Gender Bias in der Gesundheitsforschung: Deutsche Bearbeitung eines vom kanadischen Gesundheitsministerium herausgegebenen Handbuchs, erarbeitet von Margrit Eichler et al. Dezember 1999. Berliner Zentrum für Public Health; 2002. Accessed 25 Jun 2021.

Clow B, Pederson A, Haworth-Brockmann M, Bernier J. Rising to the Challenge: Sex- and gender-based analysis for health planning, policy and research in National Collaborating Centre for Determinants of Health. 2009. https://nccdh.ca/resources/entry/rising-to-the-challenge. Accessed 25 Jun 2021.

Day S, Mason R, Lagosky S, Rochon PA. Integrating and evaluating sex and gender in health research. Health Res Policy Syst. 2016; 14(1):75. doi:10.1186/s12961-016-0147-7.

Day S, Mason R, Tannenbaum C, Rochon PA. Essential metrics for assessing sex & gender integration in health research proposals involving human participants. PLoS One. 2017; 12(8):e0182812. doi:10.1371/journal.pone.0182812.

DFG. Die „Forschungsorientierten Gleichstellungsstandards“ der DFG: Zusammenfassung und Empfehlungen 2020: 1) Rekrutierungsverfahren zur Gewinnung von Wissenschaftlerinnen 2) Entlastung von Wissenschaftlerinnen für die Gremienarbeit 2020; 1–49. https://www.dfg.de/de/grundlagen-rahmenbedingungen/grundlagen-und-prinzipien-der-foerderung/chancengleichheit/allg-informationen/gleichstellungsstandards. Accessed 20 Dec 2023.

Döring N. Zur Operationalisierung von Geschlecht im Fragebogen: Probleme und Lösungsansätze aus Sicht von Mess-, Umfrage-, Gender- und Queer-Theorie. GENDER. 2013;5:17–8. https://elibrary.utb.de/doi/abs/10.3224/gender.v5i2.09. Accessed 25 Jun 2021.

Doull M, Welch V, Puil L, Runnels V, Coen SE, Shea B, et al. Development and evaluation of 'briefing notes' as a novel knowledge translation tool to aid the implementation of sex/gender analysis in systematic reviews: a pilot study. PLoS One. 2014;9:e110786. doi:10.1371/journal.pone.0110786.

Doyal L. Sex und Gender: Fünf Herausforderungen für Epidemiologinnen und Epidemiologen. [Sex and gender: five challenges for epidemiologists]. Gesundheitswesen. 2004;66:153–7. doi:10.1055/s-2004-813040.

Eichler M, Burke MA. The BIAS FREE Framework: a new analytical tool for global health research. Can J Public Health. 2006;97:63–8. doi:10.1007/BF03405218.

Eichler M, Fuchs J, Maschewsky-Schneider U. Richtlinien zur Vermeidung von Gender Bias in der Gesundheitsforschung. J Public Health. 2000;8:293–310. doi:10.1007/BF02955909.

Elm E von, Altman DG, Egger M, Pocock SJ, Gøtzsche PC, Vandenbroucke JP. Das Strengthening the Reporting of Observational Studies in Epidemiology (STROBE-) Statement. [The Strengthening the Reporting of Observational Studies in Epidemiology (STROBE) statement: guidelines for reporting of observational studies]. Internist (Berl). 2008;49:688–93. doi:10.1007/s00108-008-2138-4.

European Commission. Toolkit Gender in EU-funded research. 2011. https://op.europa.eu/de/publication-detail/-/publication/c17a4eba-49ab-40f1-bb7b-bb6faaf8dec8. Accessed 25 Jun 2021.

European Commission. Gendered Innovations: How Gender Analysis Contributes to Research: Report of the Expert Group 'Innovation through Gender'. 2013. https://data.europa.eu/doi/10.2777/11868. Accessed 20 Dec 2023.

European Commission. Gendered Innovations 2: How Inclusive Analysis Contributes to Research and Innovation: Policy Review: European Commission. Directorate General for Research and Innovation. 2020. https://data.europa.eu/doi/10.2777/316197. Accessed 20 Dec 2023.

Hammarström A. A Tool for Developing Gender Research in Medicine: Examples from the Medical Literature on Work Life. Gender Medicine 2007; 4:S123-S132. doi:10.1016/S1550-8579(07)80053-2.

Hammarström A, Wiklund M, Stålnacke B-M, Lehti A, Haukenes I, Fjellman-Wiklund A. Developing a Tool for Increasing the Awareness about Gendered and Intersectional Processes in the Clinical Assessment of Patients--A Study of Pain Rehabilitation. PLoS One 2016; 11(4):e0152735. doi:10.1371/journal.pone.0152735.

Hankivsky O. Women's health, men's health, and gender and health: implications of intersectionality. Soc Sci Med. 2012;74:1712–20. doi:10.1016/j.socscimed.2011.11.029.

Heidari S, Babor TF, Castro P de, Tort S, Curno M. Sex and Gender Equity in Research: rationale for the SAGER guidelines and recommended use. Res Integr Peer Rev. 2016;1:2. doi:10.1186/s41073-016-0007-6.

Hoffmann W, Latza U, Baumeister SE, Brünger M, Buttmann-Schweiger N, Hardt J, et al. Guidelines and recommendations for ensuring Good Epidemiological Practice (GEP): a guideline developed by the German Society for Epidemiology. Eur J Epidemiol. 2019;34:301–17. doi:10.1007/s10654-019-00500-x.

Jahn I. Die Berücksichtigung der Geschlechterperspektive: Neue Chancen für Qualitätsverbesserungen in Epidemiologie und Gesundheitsforschung. [Taking into consideration gender and sex. New chances to improve the quality of epidemiological and health research]. Bundesgesundheitsblatt Gesundheitsforschung Gesundheitsschutz. 2005;48:287–95. doi:10.1007/s00103-004-0993-2.

Jahn I. Geschlechtergerechte Gesundheitsforschung - Aktuelle Vorgaben der National Institutes of Health in den USA können neuen Schwung auch nach Deutschland bringen - Diskussionsbeitrag. [Sex/Gender-Responsive Health Research: New Funding Guidelines of the USA National Institutes of Health Can Bring a New Impetus to Germany]. Gesundheitswesen. 2016;78:469–72. doi:10.1055/s-0042-106647.

Johnson JL, Greaves L, Repta R. Better science with sex and gender: Facilitating the use of a sex and gender-based analysis in health research. Int J Equity Health. 2009;8:14. doi:10.1186/1475-9276-8-14.

Johnson JL, Repta R. Sex and Gender: Beyond the Binaries. In: Oliffe JL, Greaves L, editors. Designing and conducting gender, sex, and health research. Thousand Oaks: SAGE Publications, Inc; 2012. p. 17–38. doi:10.4135/9781452230610.n2.

Johnson JL, Repta R, Kalyan S. Implications of Sex and Gender for Health Research: From Concepts to Study Design. In: Oliffe JL, Greaves L, editors. Designing and conducting gender, sex, and health research. Thousand Oaks: SAGE Publications, Inc; 2012. p. 39–64. doi:10.4135/9781452230610.n3.

Klinge I. Gender perspectives in European research. Pharmacol Res. 2008;58:183–9. doi:10.1016/j.phrs.2008.07.011.

Klinge I. Sex and gender in biomedicine: promises for women and men.: How incorporation of sex and gender in research will lead to a better health care. In: Klinge I, Wiesemann C, editors. Sex and Gender in Biomedicine: Theories, Methodologies, Results. Göttingen: Göttingen University Press; 2010. p. 15–32.

Ladd AL. Gendered Innovations in Orthopaedic Science: Sex, Lies, and Stereotype: In Praise of the Systematic Review. Clin Orthop Relat Res. 2016;474:27–30. doi:10.1007/s11999-015-4577-2.

Lawrence K, Rieder A. Methodologic and ethical ramifications of sex and gender differences in public health research. Gender Medicine. 2007;4:S96-S105. doi:10.1016/S1550-8579(07)80050-7.

Leopold SS, Beadling L, Dobbs MB, Gebhardt MC, Lotke PA, Manner PA, et al. Fairness to all: gender and sex in scientific reporting. Clin Orthop Relat Res. 2014;472:391–2. doi:10.1007/s11999-013-3397-5.

McGregor AJ, Hasnain M, Sandberg K, Morrison MF, Berlin M, Trott J. How to study the impact of sex and gender in medical research: a review of resources. Biol Sex Differ. 2016;7:46. doi:10.1186/s13293-016-0099-1.

Nielsen MW, Stefanick ML, Peragine D, Neilands TB, Ioannidis JPA, Pilote L, et al. Gender-related variables for health research. Biol Sex Differ. 2021;12:23. doi:10.1186/s13293-021-00366-3.

Nieuwenhoven L, Klinge I. Gender awakening tool / bibliography: sex & gender in research | Canadian Women's Health Network. 2007. https://cwhn.ca/en/node/43342. Accessed 25 Jun 2021.

Nieuwenhoven L, Klinge I. Scientific excellence in applying sex- and gender-sensitive methods in biomedical and health research. J Womens Health (Larchmt). 2010;19:313–21. doi:10.1089/jwh.2008.1156.

NIH. Consideration of Sex as a Biological Variable in NIH-funded Research. 2015. https://www.genderportal.eu/resources/consideration-sex-biological-variable-nih-funded-research. Accessed 25 Jun 2021.

Rich-Edwards JW, Kaiser UB, Chen GL, Manson JE, Goldstein JM. Sex and Gender Differences Research Design for Basic, Clinical, and Population Studies: Essentials for Investigators. Endocr Rev. 2018;39:424–39. doi:10.1210/er.2017-00246.

Rippon G, Jordan-Young R, Kaiser A, Fine C. Recommendations for sex/gender neuroimaging research: key principles and implications for research design, analysis, and interpretation. Front Hum Neurosci. 2014;8:650. doi:10.3389/fnhum.2014.00650.

Ritz SA, Antle DM, Côté J, Deroy K, Fraleigh N, Messing K, et al. First steps for integrating sex and gender considerations into basic experimental biomedical research. FASEB J. 2014;28:4–13. doi:10.1096/fj.13-233395.

Runnels V, Tudiver S, Doull M, Boscoe M. The challenges of including sex/gender analysis in systematic reviews: a qualitative survey. Syst Rev. 2014;3:33. doi:10.1186/2046-4053-3-33.

Schiebinger L, Klinge I. Gendered innovation in health and medicine. GENDER – Zeitschrift für Geschlecht, Kultur und Gesellschaft. 2015;7:29–50. doi:10.3224/gender.v7i2.19311.

Schiebinger L, Schraudner M. Interdisciplinary Approaches to Achieving Gendered Innovations in Science, Medicine, and Engineering. Interdisciplinary Science Reviews. 2011;36:154–67. doi:10.1179/030801811X13013181961518.

Sex/Gender Methods Group. Addressing Sex and Gender in Systematic Reviews: Briefing Note. 2014. https://webcache.googleusercontent.com/search?q=cache:l8HpP-9q3C0J:https://methods.cochrane.org/sites/methods.cochrane.org.equity/files/public/uploads/KTBriefingNote_MSKFINAL.pdf+&cd=1&hl=de&ct=clnk&gl=de&client=firefox-b-d. Accessed 25 Jun 2021.

Smiler AP, Epstein M. Measuring Gender: Options and Issues. In: Chrisler JC, McCreary DR, editors. Handbook of gender research in psychology. New York, London: Springer; 2010. p. 133–157. doi:10.1007/978-1-4419-1465-1_7.

Springer KW, Mager Stellman J, Jordan-Young RM. Beyond a catalogue of differences: a theoretical frame and good practice guidelines for researching sex/gender in human health. Soc Sci Med. 2012;74:1817–24. doi:10.1016/j.socscimed.2011.05.033.

Tadiri CP, Raparelli V, Abrahamowicz M, Kautzy-Willer A, Kublickiene K, Herrero M-T, et al. Methods for prospectively incorporating gender into health sciences research. J Clin Epidemiol. 2021;129:191–7. doi:10.1016/j.jclinepi.2020.08.018.

Tannenbaum C, Greaves L, Graham ID. Why sex and gender matter in implementation research. BMC Med Res Methodol. 2016;16:145. doi:10.1186/s12874-016-0247-7.

Tomás C, Yago T, Eguiluz M, Samitier MAL, Oliveros T, Palacios G. A tool to assess sex-gender when selecting health research projects. Aten Primaria. 2015;47:220–7. doi:10.1016/j.aprim.2014.05.010.

Victorian Government. Gender and diversity lens for health and human services Victorian Women’s Health and Wellbeing Strategy: Stage Two 2006–2010. 2008. www.health.vic.gov.au/vwhp. Accessed 20 Dec 2023.

WHO. Gender analysis in health: a review of selected tools: World Health Organization; 2002. Available from: URL: https://apps.who.int/iris/handle/10665/42600. Accessed 20 Dec 2023.

WHO. Incorporating intersectional gender analysis into research on infectious diseases of poverty: a toolkit for health researchers. Geneva, Switzerland: World Health Organization; 2020.

Zeitler JUS. Sex/Gender equitable healthcare: Attention, challenges, and recommendations for a sex and gender sensitive approach in guideline development- using the example of German National Disease Management Guidelines [Dissertation]. Osnabrück: Universität Osnabrück; 2018.
